# Connecting, learning, supporting: Caregivers’ experiences of a stress and distress biopsychosocial group intervention

**DOI:** 10.1177/14713012231207946

**Published:** 2023-10-27

**Authors:** Craig F Wilson, Sue Turnbull, Lisa Gadon

**Affiliations:** Institute of Health and Wellbeing, 3526 University of Glasgow, UK; Psychological Therapies for Older People Service, 3077 NHS Lanarkshire, UK

**Keywords:** young-onset dementia, stress and distress, caregiver intervention, group intervention, qualitative research, biopsychosocial intervention

## Abstract

**Background:**

Family caregivers are fundamental in supporting people living with dementia to remain at home, however, psychological distress can occur as a result of their caring role. Research into interventions for caregivers of people living with young-onset dementia, including their experience of and the mediating processes of such interventions, remains limited.

**Methods:**

An Interpretative Phenomenological Analysis explored caregiver experiences and influence on caregiving of participating in a “Responding to Distress in Dementia” group. Five family caregivers were interviewed with discussions covering the period from first noticing symptoms to the interview session.

**Results:**

Within the group experience, four superordinate themes were identified: *‘connecting to other caregivers’*, ‘l*earning about caregiving’*, ‘*group factors’* and ‘*reduced caregiver distress’*. During the post-group period, three superordinate themes were recognised: *‘maintaining support’*, *‘applying learning’*, and *‘normalising caregiving’.*

**Conclusions:**

The study highlighted several interrelated themes involving creating connections amongst caregivers with similar experiences, social learning, and supportive learning through group structure and facilitation. Many of the processes reflected those found in existing dementia caregiver intervention research. Recommendations included facilitating peer support groups and exploring whole-family approaches.

## Introduction

Two-thirds of people living with dementia live at home (Livingston et al., 2014) with families often providing care. This caregiving role can impact wellbeing, leading to difficulties with mental and physical health (Brodaty & Berman, 2008). The Scottish Government has recognised the importance of supporting caregivers (Scottish Government, 2010); however, much of the research investigating the impact of caregiving focuses on those diagnosed after the age of 65 with less focus on those diagnosed prior to the age of 65.

Although there are similarities in the experiences of both caregiving groups (Roach et al., 2008), there are specific issues for caregivers of those living with young-onset dementia. Families are more likely to face financial and employment difficulties (van Vliet et al., 2010) as the condition occurs whilst more people are of working age. Parents may be supporting dependents living at home who can find the change in relationships distressing (Roach et al., 2008), and young people’s future prospects may be compromised due to taking on supporting roles (Spreadbury & Kipps, 2019). Frontotemporal dementia is more common in young-onset dementia (Viera et al., 2013), alongside more severe and pronounced behavioural changes (Mendez, 2006). Thus, when young-onset dementia caregivers attend post-diagnostic support groups, they may feel out of place or that the service cannot meet their specific needs (Giebel et al., 2020); or may require adaptations such as online facilitation to access support (Bannon et al., 2022).

Ongoing research in young-onset dementia focuses on understanding experiences of those living with dementia, including family caregivers. A systematic review into the experiences of people with young-onset dementia and their families (Spreadbury & Kipps, 2019) highlighted the challenges faced by caregivers from initial symptom onset through to after diagnosis. Initially, caregivers had difficulty identifying symptoms of dementia, attributing changes in their family member to psychosocial difficulties (Ducharme et al., 2013; Wawrziczny et al., 2016). Diagnosis was often lengthy (Bannon et al., 2022; Carter et al., 2018; Sansoni et al., 2016; van Vliet et al., 2013), with uncertainty and misdiagnosis (Mendez, 2006) contributing to distress. Adjustment to diagnosis involved information seeking about young-onset dementia (Millenaar et al., 2014), managing financial changes (van Vliet et al., 2010) and navigating changing family roles (Harris & Keady, 2004); all whilst balancing employment and parenting. Feelings of burden, concerns about the future, and feelings of loss were reported (Spreadbury & Kipps, 2016, 2019). These experiences emphasise the need to support young-onset dementia caregivers.

Interventions for dementia caregivers aim to provide knowledge, understanding, and skills for facilitating caregiving. Several reviews (e.g. Wiegelmann et al., 2021) and meta-reviews involving dementia caregivers (Dickinson et al., 2017; Gilhooly et al., 2016) support the use of psychosocial interventions in reducing adverse effects of caregiving, however, limitations remain in the evidence base such as small sample sizes, and variations in group content. As studies did not differentiate between caregiver samples, it is difficult to ascertain the benefits specifically for young-onset dementia caregivers. Recent research has, however, indicated benefits for young-onset dementia caregivers using individual online (Metcalfe et al., 2018) and home-based couple approaches (Larochette et al., 2020).

Quantitative studies, in mixed dementia populations, have been limited in identifying specific aspects within interventions that alleviate the adverse effects of caregiving. This is partly due to complexity of interventions (Sommerlad et al., 2014). In a systematic review of UK-based interventions for non-condition-specific caregivers, discussing the caregiving role whilst being recognised as a caregiver; and experiences being validated and normalised helped facilitate positive outcomes (Victor, 2009). Research has implicated processes including the change from emotion-focused to problem-focused strategies (Lockeridge & Simpson, 2012); caregiver attitudes (acceptance of the diagnosis and caregiving); access to therapists; and the use of cognitive reattribution techniques (Sommerlad et al., 2014) as beneficial. Multi-component interventions incorporating educational and therapeutic elements, and group delivery were the most widely adopted practices (Dickinson et al., 2017).

Research has primarily focused on later-onset dementia populations and it cannot be assumed that similar interventions will necessarily be beneficial for younger caregivers. A recent meta-synthesis (Bannon et al., 2022) of support-service use from a young-onset dementia perspective highlighted that caregivers wanted education about self-care approaches; and to engage in support via flexible approaches such as online groups. Studies have also highlighted benefits from psychoeducation and therapeutic skills-building interventions (Larochette et al., 2020; Metcalfe et al., 2019). Further research is required to evaluate caregiver interventions and the processes within these interventions in the young-onset dementia caregiver population.

### Research aims

The present study aimed to explore how young-onset dementia caregivers experienced a psycho-educational group intervention. Of particular interest were the experiences and processes within the group that participants perceived as being influential in their caregiving.

## Methods

### Participants

Five participants were recruited from two in-person “Responding to Distress in Dementia” (Thurlby et al., 2013) caregiver groups, delivered by two specialist nurses in a National Health Service (NHS) Young-Onset Dementia service. Groups were completed over a 5-month period prior to the COVID pandemic within a community hospital. Participants had to have been a family member providing care to the person living with young-onset dementia; have completed a group within the past 12 months; have attended at least four sessions; have adequate command of spoken English; and have been over 16 years old. To reduce bias, participants were excluded if they had been in a group facilitated by the researcher. This convenience sampling approach was chosen to capture recent caregivers who would recall the group. Caregivers were provided with study information and consented to be contacted to arrange an interview at the end of the intervention or during a routine post-diagnostic follow-up appointment. Consent was reconfirmed at interview. Attempts were made to recruit all 15 people who took part in eligible groups and five consented to participate. Reasons for non-participation included having had no further contact with the young-onset dementia service or not responding to recruitment correspondence. Recruitment stopped after the fifth interview as new insights were limited in the fourth interview and no further insights were evident in the fifth interview, indicating that the study had reached data saturation (Smith et al., 2009).

All participants cared for an immediate family member at home and supported activities of daily living. All caregivers received support from family, health and/or third-sector services. All caregivers had provided care for at least two years and, despite some being in employment, none worked full-time. Four participants were spousal caregivers; one a daughter. The individuals living with dementia were aged 55–64 years of age and received their diagnoses 1–20 months before interview. Symptom onset spanned 2–10 years. A summary of participant characteristics is provided in Table 1.

**Table 1.** Participant characteristics.

ID*	Age	Relationship to person with dementia	Dementia diagnosis	Estimated times		
				Diagnosis	Caregiving prior to diagnosis	Symptom onset
Adele	28	Daughter	Frontotemporal dementia	1 month	5 years	5–6 years
Beatrice	55	Wife	Alzheimer’s disease	1½ years	1 year	2½ years
Chris	58	Husband	Alzheimer’s disease	1½ years	6 months	2 years
David	67	Husband	Dementia NOS	1 year	9 years	10 years
Edith	64	Wife	Primary progressive aphasia	1 year	3 years	4 years

### Intervention

The “Responding to Distress in Dementia” group was created by NHS Scotland clinicians to support caregivers to manage their own, and the person living with dementia’s, distress (Thurlby et al., 2013). The group was designed to be applicable to caregivers living with someone with older- or younger-onset dementia. Content was informed by the evidence base surrounding ‘personhood’ (Kitwood, 1997) and the biopsychosocial model of distress in dementia (James, 2011); and included psycho-educational, skill-based and experiential components. Table 2 summarises session content. Groups were conducted in seven 90-min weekly sessions following a structured approach, for up to 10 caregivers. Caregivers completed pre-reading from a group resource booklet to facilitate discussions. Sessions included presentations based on the booklet topic interspersed with topic-led and caregiver-led discussions. Some sessions included skills development and caregivers were asked to practice and reflect on these in the subsequent session.

**Table 2.** Session overview.

Session	Content
1	Defining “Stress & Distress” behaviours in dementia
	Decision tree: Identifying who is distressed and when to intervene
2	Causes of “Stress & Distress”: The biopsychosocial model
3	Recognising caregiver distress & burnout
	Identifying “Non-Negotiables”
	Identifying caregiver qualities and skills
4	Communication in dementia
	Maintaining independence
	Pleasant events scheduling
	Environmental changes to reduce distress
5	How to respond to biological factors of distress
6	Caregiver coping strategies
7	Group review

### Design

A qualitative approach was employed to explore and make sense of caregivers’ experiences (Smith, 2015). Interpretative Phenomenological Analysis (IPA; Smith et al., 2009) was viewed as appropriate as it sought to gather data from reflective personal accounts from an individual’s understanding of, and involvement in, their experiences (Smith, 2011).

### Procedure

Semi-structured interviews lasting 60–80 min were conducted by the main author. The interview schedule (see supplementary file) was developed in consultation with the young-onset dementia team to gather data as to the group experiences and processes that participants perceived as being influential in their caregiving role. In line with guidance on IPA, this schedule was used flexibly (Smith et al., 2009) to enable experiences to be thoroughly explored (Smith & Osborne, 2015).

### Data analysis

Interviews were transcribed and analysed by the main author. Contextual, linguistic, and conceptual aspects of the data were generated through this textual analysis, and emergent themes were developed by mapping inter-relationships and differences amongst the data. The emergent themes were organised as to whether they referred to experiences before, during, or after the group. Finally, themes were grouped based on shared characteristics between emergent themes. The thematic dataset was combined and the process of developing common themes was repeated, generating superordinate and subordinate themes. Data saturation was considered when consistent commonalities and limited additional themes emerged during the analysis (Smith et al., 2009). Two transcripts were reviewed by the two co-authors to verify the accuracy of the transcription (Yardley, 2000), and to assess the validity of emergent themes.

In IPA, consideration is given to the interpretations of the researcher (Smith & Osborne, 2015) in understanding the participants’ lived experiences. The main author was a clinical psychologist in training on placement within the young-onset dementia service, had previously co-facilitated the group, and had delivered an intervention using the group resources. These experiences were reflected on during the research process. The main author completed a reflective diary prior to analysis, which was used during analysis and in conversations with the co-authors to ensure transparency and differentiation between the researcher’s assumptions and the participants’ experiences.

### Ethics

Ethical approval was sought and granted by the West of Scotland Research Ethics Committee and site management approval was granted by the host NHS Research and Development team.

## Results

**Figure 1.**
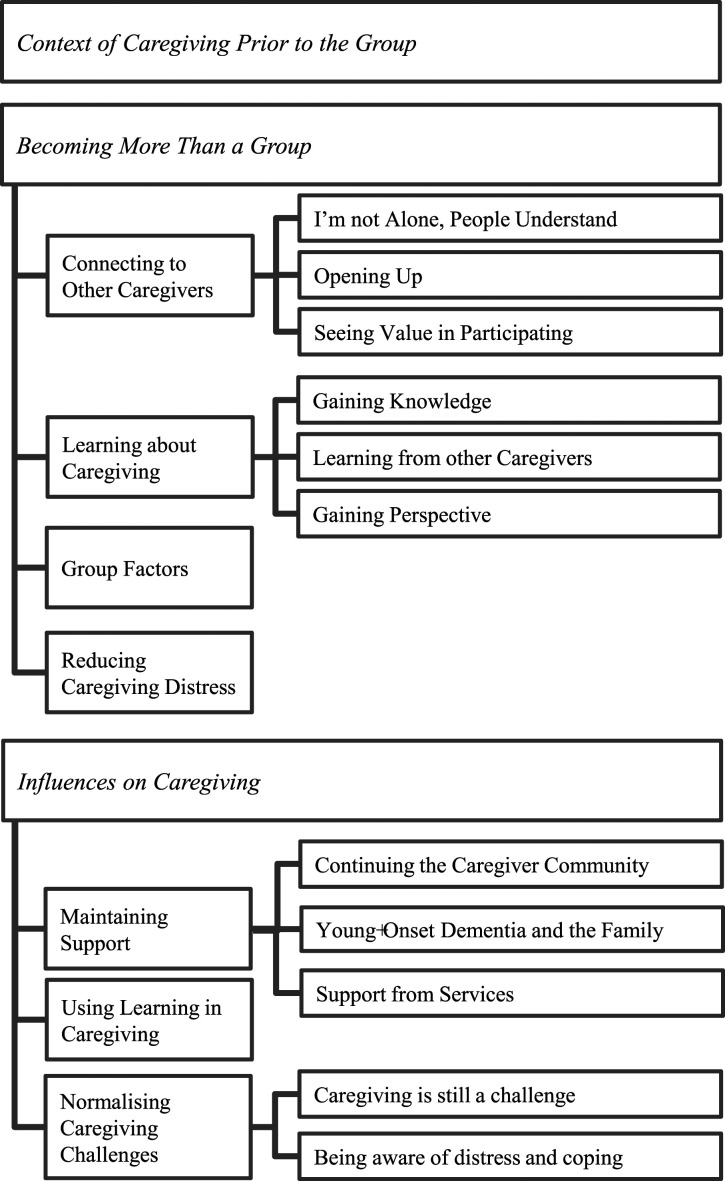
Diagrammatic representation of superordinate and subordinate themes.

Interviews highlighted experiences of the whole caregiving journey, encompassing pre-group, during the group, and post-group. Pre-group experiences are presented briefly to provide context for understanding experiences within the group and in post-group caregiving. The main analysis focused on during-group and post-group experiences and generated seven interrelated superordinate themes, and several subordinate themes presented in Figure 1.

### Context of caregiving prior to the group

All participants described difficulties within the diagnostic journey and post-diagnosis, including isolation, a lack of support and understanding, and caregiver distress. Every participant described loneliness resulting from not knowing others caring for family with young-onset dementia; and some reported that friends, family and the general public did not understand young-onset dementia. Four participants described feeling deskilled despite having prior caregiving experiences. David found that people avoided discussing his wife’s dementia, furthering his sense of isolation. All participants described challenges in finding age-appropriate support when symptoms first presented; and highlighted difficulties in obtaining a diagnosis, with symptoms misattributed to stress (Chris) and depression (Edith) by general practitioner doctors.

Participants focused on caregiving at the cost of their own self-care, Edith remarked that *“*dementia takes over everything”. Three participants expressed attempting to source information but found it confusing and, at times, distressing. Four participants reported emotional difficulties including anxiety, depression, and low self-esteem. Feelings of loss were reported by two participants. Difficulties also affected wider family with participants expressing hesitance in asking for support from family because of their own difficulties coming to terms with the situation.

### Exploring the group experience: Becoming more than a group

Themes reflected the processes of becoming connected to other caregivers and engaging in the process of learning, leading to reduced distress. Interviews also identified factors directly related to group structure, content and facilitation.

#### Connecting to other caregivers

The importance of caregivers coming together was highlighted in three subthemes: *‘I’m not alone, people understand*’, ‘*opening up*’, and ‘*seeing the value in participating*’.

##### I’m not alone, people understand

“...with my dad being ill for so long we’ve had no support before, so coming to the group was just… amazing to hear we’re not alone…” (Adele)

Every participant highlighted no longer feeling alone, emphasising relief at finding others in the same situation. Edith explained “you read in books that there’s thousands of people like you”, expressing the difference between ‘knowing’ and the physical presence of others. For Adele, being with another adult child of a parent with dementia was helpful. Participants used metaphors such as being in the same “boat” (Beatrice), “level” (Chris), and “planet” (David) to indicate feeling understood. Also of importance was a non-judgmental, validating experience:

“... other people understand what you’re saying ‘cause you can just say what you feel and nobody’s gonnae judge or... or you don’t feel stupid…” (Adele)

##### Opening up

“... I’d just sit and start speaking if you know what I mean just from the heart, from the mind…” (David)

Chris and David described speaking after recognising commonalities in caregiving experiences. All participants were able to share their thoughts, feelings and personal experiences. The ‘release’ of worries and emotions was reported, reinforcing the sense of community:

“… reached that stage of being able to open up, and sometimes opened up and got, quite emotional about it. It was good, because you need that, release” (Chris)

Openness was facilitated by having space to talk about their situation, which rarely occurred outside the group; however, three caregivers indicated withholding some information. For example, David indicated that some strategies no longer worked but *“*didn’t want to discourage anyone”.

##### Seeing value in participating

Caregivers spoke about value in participating. This allowed Edith to recognise the importance of self-care:

“…listening to them… all the wee things that they would do and, and I thought ‘you know I’m gonnae enjoy this and I’m gonnae take time’…” (Edith)

Participants valued hearing from each other, creating a new support network in the process

“...you develop this sorta, rapport, with… sort of good friendship going on with each other, mateyness sort of thing, and care for them also…” (David)

Collective nouns such as “clan” (David) and “pals” (Beatrice) emphasised cohesion and mutual respect in the group.

#### Learning about caregiving

This superordinate theme reflected the impact learning had during the group in three subthemes: *‘gaining knowledge’*, *‘learning from other caregivers’*; and *‘gaining perspective’*.

##### Gaining knowledge

“… you need an expert to explain to you really what’s going on really… I was looking for clarity and understanding and knowledge, and I think I found some of that there” (David)

Participants learned about symptoms of dementia and distress behaviours from the booklets and content-directed discussion supported by facilitators. This allowed Adele and Beatrice to understand distress caused by symptoms such as hallucinations. The written materials were cited by participants, with Adele finding benefit from “them going through the booklets with you” during sessions, and others noting being able to review the book later:

“… there was something in the book… and then it happened at home, it happened with [Husband] and I thought ‘oh that’s alright’, I think it was the... hallucinations...” (Beatrice)

There was reference to the use of cognitive strategies included in the resources:

“when I feel stressed and I think ‘what am I worrying about?’ [chuckled] ‘does it matter does it matter really?’” (Chris)

##### Learning from other caregivers

Learning from each other, stimulated by information in resource booklets and sessions, was highlighted as beneficial. All caregivers described sharing care approaches, for example, Chris learned about breaking tasks into chunks from another caregiver. Participants valued peer-learning, encouragement and support, demonstrating an implicit motivation for mutual support:

“… it was the case of, ‘well what can I offer to help you? What can you tell me that helps me?’, and that was when the support really began to work” (Chris)

Some participants explained finding conversations difficult as they faced the progressive nature of dementia:

“…how bad he’s gonnae get or how bad he could get… I’d say maybe in the back of ma head I knew that was going to happen I just I didn’t want tae, admit it to myself” (Beatrice)

By discussing difficult situations, caregivers were supported by their peers. Chris experienced difficulty when discussing violent behaviour, but recognised that information provided “an encompassing view” of a range of presentations.

##### Gaining perspective

Comparisons between experiences enhanced understanding of caregiving. David’s use of the metaphor “different bus stops, different routes” highlighted the progressive nature of dementia and differences in dementia subtypes between caregivers. Perspective-taking provided a sense of relief and opportunities to gain support with future difficulties. Chris identified that by sharing, “the penny drops for you”, indicating the enhanced understanding of personal situations through reflection:

“…sometimes you feel guilty that you’re not there all the time... they explained like that you can’t be there all the time...” (Adele)

By discussing assumptions about caregiving, participants reappraised their beliefs about caregiving situations. This occurred for Edith, when another caregiver’s expression of commitment to self-care allowed her to engage in self-care and changed her beliefs about this being selfish. For Beatrice, this led to an awareness of her stress: “I didn’t even realise there was a load on my shoulder”.

#### Group factors

Four participants discussed group factors. For example, Chris raised the importance of group size to facilitate everyone opening up. Edith highlighted being able to approach facilitators as helpful:

“…if you wanted to discuss anything, freely, you were, that it was okay to discuss anything you liked, but if there was something that was really bothering you, or, that was personal, you could say to the girls [facilitators] and they would take time, to, to go through that with you...” (Edith)

Space to discuss concerns they were worried would upset the family member living with dementia was important, further evidenced by requests that disclosures be acted upon by facilitators. Caregivers identified flexibility within sessions, with a balance between adhering to structured content and caregiver-driven conversations. This balance was not always experienced positively, for example, Adele voiced concerns that when she raised specific questions regarding medications staff “didn’t really go into any more detail”.

#### Reducing caregiver distress

All participants described reductions in distress. For Adele, learning strategies to support her father was helpful:

“…now we know about how-how to do things, it’s taken away so much stress…” (Adele)

Adele referred to being in the group as “therapy”, other participants also indicated reduction in their stress levels. For David, however, the timing meant he derived less benefit:

“…if I’d been there, nine years ago it probably be more beneficial then but, then again it, what works for someone else doesn’t necessarily work for you …” (David)

### Influences on caregiving following the group

Following completion of the group, interviews identified three superordinate themes reflecting the maintenance of support, the continued use of learning from the group in caregiving roles*,* and normalising caregiving.

#### Maintaining support

This highlighted continuation of support and was comprised of three subthemes reflecting ongoing support from peers, families and services.

##### Continuing the caregiving community

All participants reflected on the importance of keeping their newfound community, using social media to arrange additional meetings, which Beatrice referred to as “us time*”.* Adele highlighted “when you’ve got it you need to cling on to it”, and Chris considered the loss of connection as threatening, stating that “it would take something away from what I’d, what I’d gained”.

For four participants, knowing they were not alone was an enduring comfort. Edith reminded herself about the experience of others in her group, allowing her to normalise her situation and find ways to cope:

“… I think ‘I wonder how [Caregiver]’s getting on with her husband’… then I think ‘you know what, there’s people just, as bad off as yourself and they’re just coping with it and getting on with it, just the very same, so just, carry on and do what you’re doing’...” (Edith)

##### Young-onset dementia and the family

Following the group, some participants found new ways to involve family in support:

“we took all the booklets to all our family as well, let them read it... got them a bit of a better insight, we were going back and telling them all the different things we were now changing in the house, getting them to say like if any if my dad’s starting to do anything... “you need to let us know, this is how you document it, so’s we can try and see patterns, triggers, anything that could be”... and they all started doing that so that was a big help for us…” (Adele)

Other participants also noted changes within their family, with Beatrice’s son sourcing information from the internet, and Chris’s children asking to help. The group facilitated the beginnings of a whole family approach to providing care, however, spousal caregivers remained reluctant to ask their children for support.

##### Support from services

Access to appropriate and effective services was important to caregivers, however, with mixed experiences. Four participants explained how the young-onset dementia service provided valuable support to them.

“I know [CPN]’s there if I need... to ask questions or... a wee bit of advice…” (Beatrice)

David found a benefit from being able to offload to staff, recognising that “just speaking to someone, does help”. Conversely, one participant wished for more input but was reluctant to ask. Participants identified ongoing difficulties with non-specialist services, such as with hospital and GP staff understanding of young-onset dementia.

#### Using learning in caregiving

All participants spoke about applying learning following the group to help them to support their family member with dementia. Although there were ongoing challenges with caregiving, most were able to apply strategies learned in the group, and recognise when they were no longer suitable. For others, an enhanced understanding of dementia helped them cope with changes in their family member’s condition.

“it’s funny cause I didn’t panic ... I just knew... that there was, part of the Alzheimer’s because they had mentioned it at the group and that I wouldn’t have known otherwise” (Beatrice)

Participants used practical and cognitive strategies introduced in the group to help manage situations, for example, using sayings or ‘catchphrases’, such as Edith taking “ten steps back” and recognising that she is doing the best she can; using whiteboards; and using distraction:

“I knew, what ways to do it or try and use diversion tactics, I didn’t do that before I would just sit and let him go on before...” (Adele)

Ongoing learning was supported by the resources:

““the group certainly has helped me change my way of thinking because, there was a thing in one of the-the booklets that [Nurse] gave me and it was about this woman who just wanted to do everything herself… you really don’t relate to that, you don’t think you’re doing that, but I think I was maybe really guilty of doing that” (Edith)

Not all resources and strategies were found to be helpful by all caregivers, for example, when a tool did not fit with views on caregiving:

“I don’t have non-negotiables” (Chris)

#### Normalising caregiving challenges

This reflected the dichotomy between coping and continuing challenges in caregiving. Two subthemes were identified: ‘caregiving is still a challenge’ and ‘being aware of distress and coping’.

##### Caregiving is still a challenge

All participants spoke about caregiving remaining practically and emotionally demanding. Chris felt that “everyday’s a different challenge”*,* highlighting the inconsistent nature of his experience of dementia. David experienced less improvement following the group:

“…the group’s helped a lot really but it hasn’t changed my situation any, in reality I still have the same situation which’s, [Wife] becoming more dependent… I know that and I accept that…” (David)

Two participants spoke of experiencing events that had consequences on the whole family system, for example, their relative living with dementia being hospitalised.

##### Being aware of distress and coping

Four participants described feeling better able to cope following the group, both with the distress expressed by the family member living with dementia, and within themselves.

“I think when I’m stressed, I realise quicker, now, and I try... to relax” (Beatrice)

In addition, Beatrice also drew comfort from “knowing that [services] are here, and everybody that was in the group”. A commitment to self-care was noted as an aspect of ongoing coping.

“I mean, going, look after [Wife] depended on how I am, and being with the group I think has made me feel a bit better, so being in, the concept that ‘I am better than I was’ I look after her better, so, looking at it that way, yes…” (David)

Similarly, Beatrice was able to arrange the week to incorporate time to herself. These were seen as positive changes, and aided by the perspective gained from speaking to other caregivers.

## Discussion

The experiences of caregivers participating in a “Responding to Distress in Dementia” group identified four superordinate themes during the group. *‘Connecting to other caregivers’* highlighted a transition from social isolation to developing strong relationships with those with similar experiences. Being understood by a peer group with shared experiences facilitated open discourse, promoting the development of a support network and gave credibility to discussions about coping strategies. *‘Learning about caregiving’* emphasised knowledge gained about caregiving, leading to the development of coping strategies. Social learning, facilitated by group material and peer discussions, allowed caregivers to reflect and make sense of caregiving experiences, and re-appraise their expectations. *‘Group factors’* focused on specific aspects of the group that supported the intervention and included group size, effective facilitation and flexibility of group structure. Lastly, *‘Reducing caregiver distress’* recognised the benefits to the participants and personal characteristics that influenced the effects of the group.

In the post-group period, three superordinate themes were identified. *‘Maintaining support’* emphasised the continuation of practical and emotional support from peers and services, and in engaging wider family support. *‘Applying learning’* highlighted the caregivers’ continued use of strategies and understanding of dementia to help coping. Finally, *‘Normalising caregiving’* recognised ongoing challenges of caregiving and increased awareness of and response to distress. This supported engagement in coping strategies such as self-care and reaching out for support.

In the current study, *‘connecting to other caregivers’* was important and reflected evidence that caregivers benefit in sharing their difficulties outside the family (Larochette et al., 2020). This was in the context of the challenges noted during the pre-group period, such as the lack of understanding about young-onset dementia and stigma perceived by caregivers. This resonated with existing evidence of the social difficulties caregivers find themselves navigating during this time (Spreadbury & Kipps, 2016, 2019) and chimes with the social model of disability research in dementia that recognises the marginalisation of people with young-onset dementia in existing care structures (Gilliard et al., 2005). The strong peer support network developed during the groups, through shared understanding and discourse, reflects similar mechanisms of change previously identified in a systematic review of caregiver interventions (Victor, 2009).

Reducing isolation is a central theme across research with dementia caregivers (e.g., Bannon et al., 2022; Ducharme et al., 2013) and was described by all participants despite previous involvement with dementia groups. Perceived similarity between group members has been identified as an important mechanism of change in interventions (Victor, 2009), with peer support for later-onset dementia caregivers (Bunn et al., 2012, 2015), and was important to the current study’s young-onset dementia caregivers.

The commitment to *‘continuing the caregiver community’* between participants emphasised the importance of peer support, and reflected previous findings that social support is a key mechanism of change in psychosocial interventions for later-onset dementia caregivers (Elvish et al., 2013). Bannon and colleagues’ (2022) meta-synthesis indicated that caregivers value support to reduce isolation. Given the challenges caregivers have described in speaking about dementia, both in the current study and in wider research (Larochette et al., 2020), an ongoing sense of community support seems an important step to reducing isolation.

*‘Learning about caregiving’* processes identified within the group included reciprocal sharing of knowledge, learning about dementia, having access to specialist knowledge and information, and normalisation of experiences. Similar processes have been identified in later-onset dementia caregiver interventions (Sommerlad et al., 2014) and young-onset dementia service research (Carter et al., 2018; Sansoni et al., 2016). Studies have found benefits to acknowledging stress and gaining understanding through learning about dementia and caregiving (Metcalfe et al., 2018). Age-specific and disease-specific learning resources have been highlighted as important in a recent meta-synthesis (Bannon et al., 2022).

Without intervention, young-onset dementia caregivers have reported using coping strategies that lead to adjustment difficulties and negative outcomes (Lockeridge & Simpson, 2012). Although not specific to young-onset populations, interventions that reduce unhelpful coping such as denial and avoidance have been associated with improved outcomes for caregivers (Gilhooly et al., 2016). Within the present study, emotion-focused and problem-focused coping strategies were noted; and cognitive reattribution of thinking styles appeared to facilitate changes in coping style. Participants utilised emotion-focused self-disclosure within the group and learned different problem-focused strategies for their difficulties. Importantly, beliefs about self-care were addressed and they gained social permission to engage in self-care, echoing findings from Bannon et al. (2022) who identified a common theme of the importance of caregivers prioritising their own self-care.

Group size, access to comprehensive materials for future reference, and facilitation by experienced staff were key *‘group factors’*. Victor (2009) highlighted that flexibility and personalisation of interventions were important to caregivers. Although caregivers remarked on specific group resources, their focus was on the interpersonal and therapeutic components such as time to express emotions and collaboratively reframe cognitive attributions. The group also encouraged caregivers to engage in meaningful activities out-with caregiving, which has been considered helpful in reducing caregiver burden (Wiegelmann et al., 2021); however, again this was not consistently commented upon in the current study. A meta-synthesis of support preferences for those accessing young onset dementia services highlighted group factors reflected in the current study, including direct and interactive content, and flexibility in service delivery (Bannon et al., 2022).

Timing of interventions may be important in the perceived usefulness of interventions, with studies suggesting access at earlier stages of the caregiving journey being of increased benefit (Victor, 2009). In the present study, some expressed wishing to have participated sooner. Most participants experienced lengthy pre-diagnostic periods spanning years, but were offered the group within 18 months of diagnosis. Thus, these remarks may have been indicative of the difficulty of diagnosing young-onset dementia, which is known to be challenging (Carter et al., 2018; Spreadbury & Kipps, 2019). Dementia subtype did not appear to be relevant for participants in this study to feel supported by others. Within the later-onset dementia group intervention literature, there have been concerns that including people at different stages of the dementia journey can lead to distress from increased awareness of the trajectory of their loved one’s condition (Bunn et al., 2012). Although some of the current study’s participants were concerned about discussing the future, benefits from these discussions included being able to express emotions associated with fear and concern for the future within a supportive environment, with people experiencing similar life-changing events. The ability to explore emotionally difficult topics whilst being contained in a structured, socially cohesive group may have been responsible for some of the benefits described by caregivers.

### Recommendations

In line with reviews into young-onset dementia services highlighting the importance of adapting services to this population (Giebel et al., 2020; Mayrhofer et al., 2018; Stamou et al., 2021), there are a number of recommendations that can be made from the current study for adaptations to tailor group interventions for this population. The importance of social connection was noted in this study and wider literature (Elvish et al., 2013; Stamou et al., 2021), indicating that facilitating the development of peer support networks appears helpful (Giebel et al., 2020). Thus, pre- and post-group social components were included in the protocol for future groups and it will be important to evaluate the impact of this adaptation. Addressing the family dynamics around supporting caregiving may be an important addition to interventions (Stamou et al., 2021). Adult children were included in the current groups and others became more involved in caring following a parent’s participation reflecting research into the experiences of adult children which has recognised their desire to be involved (Barca et al., 2014). Thus, services should consider whole-family support approaches following the diagnosis of dementia.

The use of online resources has been found to be helpful in supporting younger-onset dementia caregivers (Metcalfe et al., 2018) and this may enable younger family members to be included. Although there is growing evidence for group-based psychosocial programmes to be included as part of routine post-diagnostic support, for people with young-onset dementia and their caregivers, age-appropriate resources continue to be fragmented and often delivered as short-term projects (Mayrhofer et al., 2018). Therefore, consideration needs to be given as to how to enable equitable access to such support. Given the adoption of online groups within the NHS during the Covid-19 pandemic, it would be useful to adapt this group online for the benefit of young-onset dementia caregivers.

### Limitations

Although the timing of the interviews allowed for reflection on the caregiving journey, the short timeframe between completion of the group and the interviews may not have allowed sufficient time for participants to fully digest their group experiences. As noted by Sommerlad et al. (2014), the complexity of group interventions meant that the significance of particular aspects of the group were difficult to determine. However, processes identified in the present study have also been demonstrated as effective in wider research.

As several potential participants either declined to take part or were unavailable as they were no longer open to the young-onset dementia service, the sample may not have gathered views of those that could not attend due to higher support needs or had experienced less benefit from the intervention. Previous research has also identified similar participant bias (Victor, 2009). Despite these concerns, some participants did express negative experiences within the group.

Sample homogeneity may have been reduced due to the inclusion of a non-spousal caregiver (daughter). However, participants’ pre-group experiences were all similar, and the use of structured content within sessions allowed for sufficient homogeneity for interpretation using IPA (Smith et al., 2009). Lastly, as there were consistent commonalities and limited additional themes being identified during analysis, the likelihood of data saturation is a relative strength in the present study.

### Conclusions

The present study explored caregiver experience of engaging in the “Responding to Distress in Dementia” group intervention for caregivers of family members with a diagnosis of young-onset dementia and considered how this transferred into and supported their caregiving role. The findings add to the growing evidence for age-appropriate group-based psychosocial programmes to be included as part of routine post-diagnostic support for people with young-onset dementia and their caregivers. They also add to existing evidence regarding the experiences of caregivers to inform future service provision. Several inter-related themes were identified as beneficial, including creating a connection to people with similar experiences, engaging in social learning, and being supported to do so through the structure and facilitation of the group. Service recommendations included a whole-family approach and the facilitation of peer support throughout the caregiving journey. With young-onset dementia interventions in their primacy, more research is required to support effective practices for this population.

## Supplemental Material

Supplemental Material - Connecting, learning, supporting: Caregivers’ experiences of a stress and distress biopsychosocial group intervention Click here for additional data file. Supplemental Material for Connecting, learning, supporting: Caregivers’ experiences of a stress and distress biopsychosocial group intervention by Craig F Wilson, Sue Turnbull and Lisa Gadon in Dementia
