# Supplementary material for: Connecting, learning, supporting: Caregivers’ experiences of a stress and distress biopsychosocial group intervention
Source: Dementia (London). 2023 Oct 27;23(1):23–40. doi: 10.1177/14713012231207946 (PMC10798010; doi:10.1177/14713012231207946)
Supplement: Supplemental Material - Connecting, learning, supporting: Caregivers’ experiences of a stress and distress biopsychosocial group intervention [file sj-pdf-1-dem-10.1177_14713012231207946.pdf]

Interview Schedule

Introducing questions: **So why don't we start by you telling me about who you are? Who do you care for? And what about the rest of your family?** Check consent forms and query.

**Focus Point: To understand carers' experiences of the group**
Intro: **I'm interested in how you experienced the group. I thought we could start by talking about what it was like for you?**<br>• **What was the group like?** How did you find it? How was it for you?<br>• **How did you find out about the group?**<br>• **What parts of the group did you like?** What made this likeable? What did you enjoy?<br>• **What about the things you didn't like?** What made this dislikeable? What did you not enjoy?<br>• **What parts of the group were you most interested in?** What did you find yourself thinking about in the group? If people were to ask you about the group, what would you say?<br>• **Is there anything you hoped for from the group that you didn't get?** Is there anything that wasn't addressed that you feel should have been? Would there be anything that would you change?<br>• **Was there anything that surprised you?** If you were talking to people about the group, what would you tell them that surprised you?<br>• **Were you able to attend all sessions? If not, what were the reasons?** Would you have liked to?<br>• **What things did you know before coming to the group?** What wasn't news to you?<br>• **How did you find talking in the group?** What did you talk about? How was it for you? Do you find talking in groups comfortable or less so?<br>• **Was there anything other people said that you found useful/agreed with/disagreed with?** What was this? What was it about that for you?<br>• **Do you feel that you participated at the right time?** When do you feel is the right time? What makes it the right/wrong time? What would you say to others that are thinking about participating?

Linking questions
• **What were you told about the purpose/aims of the group?**<br>• **What made you decide to go to the group and keep going?**

**Focus Point: To identify any influence that group participation has on carers' lives (+processes)**	**Focus Point: To identify any influence that group participation has on carers' experiences of the caregiving role (+processes)**
Intro: **Now that you've been to the group, I'd like to find out about your life now. Can you tell me about the impact the group has had?**<br>• **Have things changed for you since participating in the group?**<br>• **Has anything been worse? Has anything been better? Has anything been the same?**	Intro: **I'd like to find out about what it's like caring for [X] after being to the group. How have you found caregiving since being part of the group?**<br>• **Is this a change or is it the same as before being in the group?**<br>• **Has the group made a difference to your caring role?**<br>• **What has changed? Has anything been harder? Has anything been easier? Has anything been the same?**

**Focus Point: To identify the processes, or mechanisms, that account for these potential influential factors**
These will be found through enquiring about the above.<br>• **What do you think is the reason for these changes?** Has there been anything about being in the group that would explain these? What about outside of the group?<br>• **What reasons have things stayed the same for you?** Has there been anything, good or bad, about being in the group that would explain these? What about outside of the group?<br>• **What were the most important things about the group for you?** Are they the reasons for things being better/worse?

**Focus Point: To understand carers' perceptions of dementia following group participation.**
Intro: **Now that you've been to the group, what do you think of your family member's dementia?**<br>After the group: **Are they the same or have they changed?**<br>o If they have changed: How have they changed? What has changed your view? Both inside and outside of the group?<br>o If they haven't changed: Can you tell me more?<br>• **How do you talk to people about your family member's dementia?**<br><br>Before the group: **Thinking about what life was like before the group started, what were your thoughts on dementia?** How did you view it? Where do you think this view came from?

**Focus Point: To understand carers' perceptions of distress following group participation.**
Intro: **One aspect of the group was to help you understand and manage your own and other's distress. Now that you've been to the group, how do you understand your family member and your own distress?**<br>After the group: **Are they the same or have they changed?**<br>o If they have changed: How have they changed? What has changed your view? Both inside and outside of the group?<br>o If they haven't changed: Can you tell me more?<br>Before the group: **Thinking about what life was like before the group started, what were your thoughts on these behaviours?** How did you view it? Where do you think this view came from?

**Ending: Lastly, I would like to discuss how you feel about the support you get.**
Support:<br>• **You mentioned that [people] are around. Who provides you with support?**<br>• **How do you feel about the support provided?** What have you received?<br>• **Do you know what to do if you require further support?**

**Signposting:**
• Query input from Young-Onset Dementia service, GP, and third-sector services (e.g. Alzheimer's Scotland Dementia Link-Worker).<br>• Signpost where appropriate (e.g. Alzheimer's Scotland, Carer Network, Mental Health), including Samaritans, NHS 24 and Breathing Space.<br>• If distress present, discuss potential for researcher to follow-up with GP or Young-Onset Dementia service.

**End:**
• Clean-up question (e.g. **that's all I have to ask, have you anything else you'd like to say, final thoughts or anything we haven't covered?**)
